# *O*-GlcNAcase contributes to cognitive function in *Drosophila*

**DOI:** 10.1074/jbc.RA119.010312

**Published:** 2020-02-24

**Authors:** Villo Muha, Michaela Fenckova, Andrew T. Ferenbach, Marica Catinozzi, Ilse Eidhof, Erik Storkebaum, Annette Schenck, Daan M. F. van Aalten

**Affiliations:** ‡ Gene Regulation and Expression, School of Life Sciences, University of Dundee, Dundee DD1 5EH, United Kindom; § Department of Human Genetics, Donders Institute for Brain, Cognition and Behaviour, Radboud University Medical Center, 6525GA Nijmegen, The Netherlands; ¶ Department of Molecular Neurobiology, Donders Institute for Brain, Cognition and Behaviour and the Faculty of Science, Radboud University, 6525XZ Nijmegen, The Netherlands

**Keywords:** Drosophila, O-GlcNAcylation, neuroscience, O-linked N-acetylglucosamine (O-GlcNAc), synapse, habituation, learning, NMJ, O-GlcNAcase

## Abstract

*O*-GlcNAcylation is an abundant post-translational modification in neurons. In mice, an increase in *O*-GlcNAcylation leads to defects in hippocampal synaptic plasticity and learning. *O*-GlcNAcylation is established by two opposing enzymes: *O*-GlcNAc transferase (OGT) and *O*-GlcNAcase (OGA). To investigate the role of OGA in elementary learning, we generated catalytically inactive and precise knockout *Oga* alleles (*Oga^D133N^* and *Oga^KO^*, respectively) in *Drosophila melanogaster*. Adult *Oga^D133N^* and *Oga^KO^* flies lacking *O*-GlcNAcase activity showed locomotor phenotypes. Importantly, both *Oga* lines exhibited deficits in habituation, an evolutionarily conserved form of learning, highlighting that the requirement for *O*-GlcNAcase activity for cognitive function is preserved across species. Loss of *O*-GlcNAcase affected a number of synaptic boutons at the axon terminals of larval neuromuscular junction. Taken together, we report behavioral and neurodevelopmental phenotypes associated with *Oga* alleles and show that Oga contributes to cognition and synaptic morphology in *Drosophila*.

## Introduction

Protein *O*-GlcNAcylation, a dynamic modification of proteins with GlcNAc on serine/threonine residues, is orchestrated by two enzymes: *O*-GlcNAc transferase (OGT)^3^ and *O*-GlcNAcase (OGA). *O*-GlcNAcylation maintains cellular homeostasis by modulating translation (1), protein stability (2, 3), and subcellular localization of proteins (4, 5). Furthermore, it plays a key role in regulating transcription (6–9) and differentiation (10, 11). Although the mechanism of *O*-GlcNAcylation is highly evolutionarily conserved, from the early metazoan *Trichoplax adhaerens* to humans (12), there are considerable differences in the extent vertebrates and invertebrates tolerate alteration in protein *O*-GlcNAcylation.

OGA, the enzyme that removes the *O*-GlcNAc modification, is the product of the *MGEA5* (meningioma-expressed antigen 5) gene in vertebrates. OGA is indispensable for late embryonic development and postnatal survival of mammals (13, 14). Mouse pups lacking OGA protein show delayed development, small size, abnormality in lung histology, and perinatal lethality (13, 14). *Drosophila Oga* null mutants, however, develop normally to adulthood (15, 16), making *Drosophila* an attractive system for uncovering previously unappreciated roles of OGA.

A substantial body of evidence indicates that *O*-GlcNAcylation is crucial for normal development and function of the mammalian nervous system (17–21). In mice, increased *O*-GlcNAcylation induced by a brain-specific knockout of OGA manifested in a delay in brain development, reduced olfactory bulb size, missing anterior pituitary, and enlarged brain ventricles and revealed that OGA is required for neurogenesis (22). It has recently been established that certain mutations in the human OGT gene cause intellectual disability (23–26). The mutations are associated with reduced OGA mRNA and protein levels (23, 27, 28), suggesting that altered OGA expression may contribute to the diverse developmental and cognitive symptoms in these patients. Furthermore, recently identified SNPs in the intronic sequence of *OGA* have associated the gene with IQ and intellectual development (29), together indicating a role for OGA in human cognition.

Recent studies have demonstrated that both acute and chronic increases of protein *O*-GlcNAcylation cause hippocampus-associated learning and memory defects in mice and rats (30, 31). Despite these studies suggesting a crucial function of OGA in normal learning, our knowledge about how OGA affects cognitive ability is limited. Therefore, we investigated whether the role of OGA in learning is conserved in *Drosophila*.

OGA is a multidomain protein; it consists of an N-terminal *O*-GlcNAc hydrolase catalytic domain that belongs to the GH84 family of glycoside hydrolases (32), a middle highly disordered “stalk” domain, and a C terminus with sequence homology to histone acetyltransferases. The histone acetyltransferase domain lacks key amino acids responsible for acetyl-CoA binding (33); thus OGA only exhibits *O*-GlcNAcase enzymatic activity. However, it has never been investigated before whether OGA possesses any nonenzymatic roles. Therefore, we also developed tools to dissect enzymatic or nonenzymatic functions of Oga in normal neuronal development and cognition/learning.

We generated rationally designed catalytically inactive *Oga* (*Oga^D133N^*) and novel *Oga* knockout (*Oga^KO^*) alleles by exploiting the CRISPR/Cas9 gene editing toolbox, resulting in elevated levels of protein *O*-GlcNAcylation in homozygous flies. We discovered that a loss of *O*-GlcNAcase activity affects locomotion and causes deficits in habituation learning, thereby demonstrating a conserved role of *Drosophila Oga* in cognitive function. Additionally, we showed that synaptic bouton counts at the larval neuromuscular junctions are altered in *Oga^KO^* flies, indicating a novel role for *Oga* in synaptic development. Our phenotypic characterization of *Oga^D133N^* and *Oga^KO^* lines also revealed that the primary role of *Oga* in these processes is *O*-GlcNAcase enzyme activity.

## Results

### Genome editing of Oga results in increased protein O-GlcNAcylation

**Figure 1.**
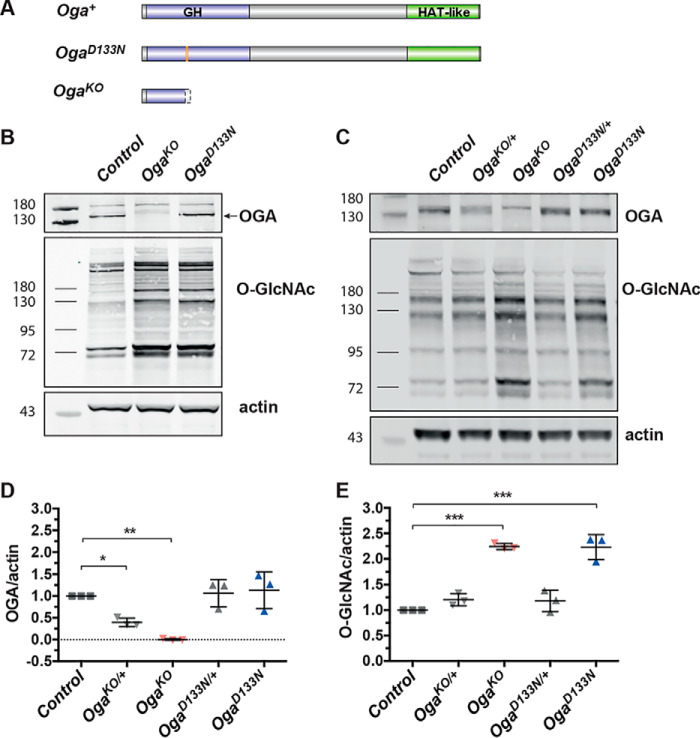
**Generation and characterization of *Oga^D133N^* and *Oga^KO^* alleles.** *A*, schematic representation of *D. melanogaster* Oga protein. *Purple*, glycosyl hydrolase (*GH*) domain; *gray*, linker domain; *green*, pseudo-histone acetyltransferase (*HAT-like*) domain. The *Oga^D133N^* allele expresses the full-length *Drosophila* Oga with a single D133N missense mutation in the GH domain (the mutation site is in *orange*). The *Oga^KO^* allele only produces a short, truncated polypeptide of 148 amino acids terminating before the catalytic core of Oga GH domain. *B*, Western blotting on 4–8-h-old *Drosophila* embryo samples indicate a lack of Oga expression in the *Oga^KO^* line and an increased level of *O*-GlcNAcylation in homozygous *Oga^KO^* and *Oga^D133N^ Drosophila* embryos. Embryos were collected from crosses of homozygous *Oga^KO^* or *Oga^D133N^* parental flies. Western blotting was probed with an anti-OGA antibody and a monoclonal anti-*O*-GlcNAc antibody (RL2), raised against *O*-GlcNAc modified nucleoporins, that recognizes a subset of the *O*-GlcNAc modified proteome. Actin was used as a loading control. A complete loss of the lower band corresponding to Oga protein was apparent in homozygous (*Oga^KO^*) samples. *C*, Western blotting on 1–4-day-old male adult head lysates was probed with an anti-OGA antibody and monoclonal anti-*O*-GlcNAc antibody (RL2). Actin was used as a loading control. The anti-OGA antibody recognizes two proteins at the molecular mass range of 130–180 kDa; the *lower band* is specific to *Drosophila* Oga, and the *upper band* is nonspecific. Complete loss of the *lower band* was apparent in homozygous *Oga^KO^* samples. *D*, quantification of Oga protein levels in adult head samples revealed that heterozygous *Oga^KO^*^/+^ flies have reduced Oga protein compared with genetic background control (one-way ANOVA with Dunnett's multiple comparisons test, *p* = 0.036, *n* = 3). Oga protein levels are unchanged in heterozygous *Oga^D133N^*^/+^ and homozygous *Oga^D133N^* samples (*p* = 0.9914 and *p* = 0.9013, respectively). Means ± S.D. are shown. *E*, quantification of *O*-GlcNAcylated proteins revealed that *Oga^KO^* and *Oga^D133N^* flies have increased *O*-GlcNAc levels compared with genetic background control *Drosophila* adult head samples (2.2-fold, one-way ANOVA with Dunnett's multiple comparisons test, *p* < 0.0001, *n* = 3 for both lines; means ± S.D. are shown).

To dissect *O*-GlcNAcase enzymatic and any nonenzymatic roles of OGA, we generated catalytically inactive and precise null *Drosophila Oga* alleles (Fig. 1*A*). The *Drosophila* Oga protein shows 57% sequence identity with the human enzyme (human OGA). Previous studies have identified a conserved aspartate, Asp^175^ in human OGA as a key catalytic amino acid in both vertebrate and bacterial OGAs (34–36). This residue is Asp^133^ in *Drosophila* Oga and, together with the rest of the catalytic machinery, is conserved throughout evolution (Fig. S1). Mutation of this aspartate to an asparagine leads to a protein species incapable of hydrolysis, yet with retention of *O*-GlcNAc protein binding (37–39). We have used a CRISPR/Cas9 gene editing approach to introduce the D133N mutation into the endogenous *Oga*, thus generating the desired catalytically inactive (*Oga^D133N^*) allele. In parallel, we isolated a null allele (*Oga^KO^*) produced by two nucleotides frameshift mutation at 3R:21219675 [+] (Oga mRNA 547 nucleotide position), resulting in a premature STOP codon and a truncated 148–amino acid–long peptide product (Fig. S2).

Homozygous *Oga^D133N^* and *Oga^KO^* animals developed to adulthood without apparent defects. To probe the effect of the newly generated *Oga* alleles on *O*-GlcNAc homeostasis, samples from *Oga^D133N^* and *Oga^KO^* embryos and adult heads were subjected to Western blotting (Fig. 1, *B* and *C*). Oga was undetectable in homozygous *Oga^KO^* samples (Fig. 1, *B* and *C*), whereas it was expressed at WT level in heterozygous *Oga^D133N^* (*Oga^D133N^*^/+^) and homozygous *Oga^D133N^* flies (Fig. 1*C*). Half gene dosage of *Oga* in *Oga^KO^*^/+^ flies led to ∼60% reduction in protein level (normalized expression: *w*^1118^ = 1, *Oga^KO^*^/+^ = 0.40 ± 0.1) (Fig. 1*D*). Protein *O*-GlcNAcylation levels were 2.2-fold elevated in samples from both homozygous *Oga^D133N^* and *Oga^KO^* flies (Fig. 1*E*). In summary, we created two new alleles of *Oga* that showed increased protein *O*-GlcNAcylation and enabled the dissection of Oga function.

### Compromised Oga function leads to a reduction in life span and locomotor defects in adult flies

**Figure 2.**
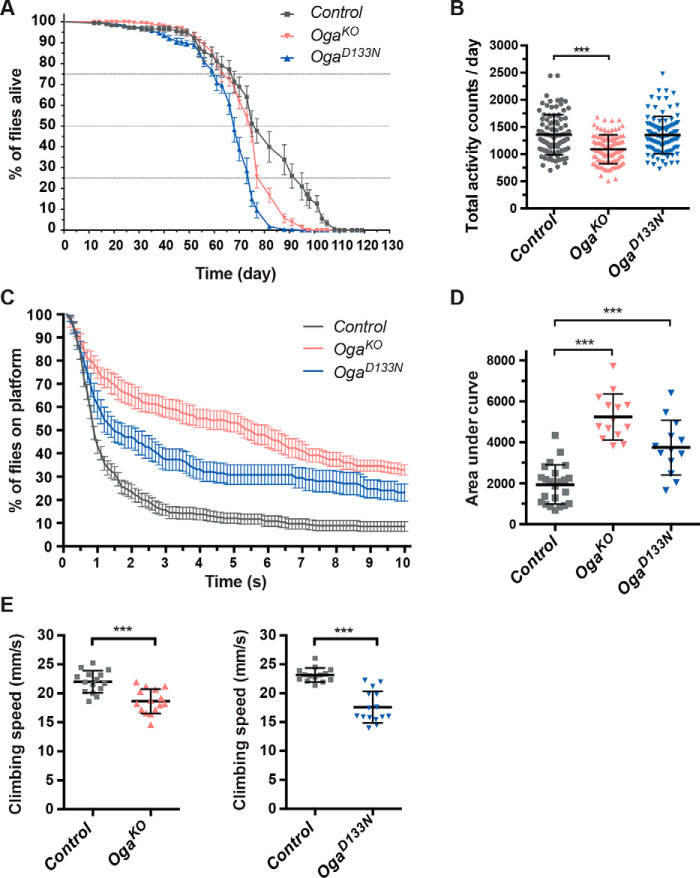
**Life span and locomotor behavior of *Oga^KO^* and *Oga^D133N^* flies.** *A*, life span of homozygous *Oga^KO^* and *Oga^D133N^* male flies. Survival of 20 flies per vial was followed until no flies were left alive. Survivorship curves show means ± S.E. of data recorded from 9–14 vials/genotype, scoring the number of flies alive every 2 days. Log-rank tests indicate decreased mean life span for *Oga^KO^* (*n* = 280) and *Oga^D133N^* (*n* = 280) flies compared with their genetic background control (*n* = 179) (control *versus Oga^KO^*, c2 = 41.5, *p* < 0.001) (control *versus Oga^D133N^*, c2 = 104.8, *p* < 0.001). *B*, total daily activity counts of control, *Oga^KO^*, and *Oga^D133N^* male *Drosophila* plotted in 12:12-h light:dark cycle. *Oga^KO^* (*n* = 101) exhibited decreased daily activity compared with control flies (*n* = 97) (*p* < 0.001, one-way ANOVA with Bonferroni's multiple comparisons test). *C*, locomotion and flight performance were assessed in the island assay. 15 flies per measurement were thrown on a white platform surrounded with water. Graphs show the percentage of flies that remain on the platform over time (10 s; means ± S.E., for control *n* = 23, *Oga^KO^ n* = 13, *Oga^D133N^ n* = 14 repeats). The data were collected over 3 days of measurement. *D*, *floating bars* depicting the mean ± S.D. AUC based on the graphs shown in *D*, one-way ANOVA with Holm–Sidak's multiple comparisons of mean AUC. Flight escape performance of *Oga^KO^* and *Oga^D133N^* flies was impaired compared with control (*Oga^KO^ p* < 0.0001; *Oga^D133N^ p* = 0.0004). *E*, climbing locomotor behavior of Oga-deficient flies was assessed based on their climbing speed (mm/s) in an automated negative geotaxis assay. *Oga^KO^* and *Oga^D133N^* groups showed significantly reduced climbing speed compared with background control, indicating locomotor dysfunction (means ± S.D.; nonparametric Mann–Whitney test, control *n* = 14, *Oga^D133N^ n* = 15, *p* < 0.0001; control *n* = 15, *Oga^KO^ n* = 15, *p* = 0.0002).

Elevated *O*-GlcNAc levels caused by high sugar and high glucosamine diet are known to shorten the median life span of adult flies (40). We first tested whether the increased protein *O*-GlcNAcylation in *Oga* mutant flies affects survival as a measure of overall health. Batches of 20 male flies collected within 24 h past eclosion were placed into vials with standard diet, and their life span was monitored for over 100 days. The mean life span for genetic background control flies was 73.0 ± 1.4 days, whereas homozygous *Oga^D133N^* (61.1 ± 0.8 days) and *Oga^KO^* (67.2 ± 0.7 days) adult males exhibited significant mean life-span reduction of approximately 12 and 6 days, respectively (Fig. 2*A*).

Loss of OGT in mouse postmitotic neurons caused a rapid increase of daily food intake accompanied with hyperactivity (21), indicating that the *O*-GlcNAc system potentially influences locomotor activity of an organism. Therefore, we tested daily locomotor activity in *Oga^D133N^* and *Oga^KO^* male adults using DAM2 *Drosophila* activity monitors. Under 12:12 h of light:dark cycle conditions, the *Oga^KO^* group consistently showed a modest but significant decrease in total daily activity counts (means ± S.D., 1088 ± 266 total activity counts/day) compared with control (1359 ± 371) and *Oga^D133N^* (1350 ± 345) groups (Fig. 2*B* and Fig. S3, *A–C*). Furthermore, the *Oga^KO^* flies exhibited a reduction in activity counts while awake (Fig. S3, *D–F*), suggesting that the observed lower daily activity is possibly associated with motor defects.

Hence, we next further explored motor phenotypes in *Oga^D133N^* and *Oga^KO^* flies using island test and negative geotaxis assays. Both tests are widely used to investigate neuronal impairment and muscular defects in *Drosophila* models of intellectual disability, neurodegeneration, and neuromuscular diseases (41–44). The island test measures escape responses requiring activation and coordination of leg and wing movements. In this assay, flies are thrown onto a white flat platform surrounded with water, and the time each individual spends on the platform is determined. Healthy WT flies normally exhibit an escape response and quickly fly away from the platform. Under the island assay test conditions, homozygous *Oga^D133N^* (area under curve parameter (AUC), shown as the mean ± S.D., was 3700 ± 1300) and *Oga^KO^* (5200 ± 1100) flies remained markedly longer on the platform than the background control line (1900 ± 1000) (Fig. 2, *C* and *D*).

In the negative geotaxis assay, young (2–4-day-old) male control flies climbed at a mean speed of 23 ± 5 mm/s. However, climbing performance of homozygous *Oga^D133N^* (17 ± 7 mm/s) and *Oga^KO^* (18 ± 6 mm/s) flies showed significantly reduced climbing speed compared with genetic background controls, indicating locomotor impairment (Fig. 2*E*). Taken together, these data show that compromised *Oga* function leads to reduced life span and locomotor defects in adult flies.

### Loss of Drosophila Oga and its O-GlcNAcase activity causes deficits in habituation learning

**Figure 3.**
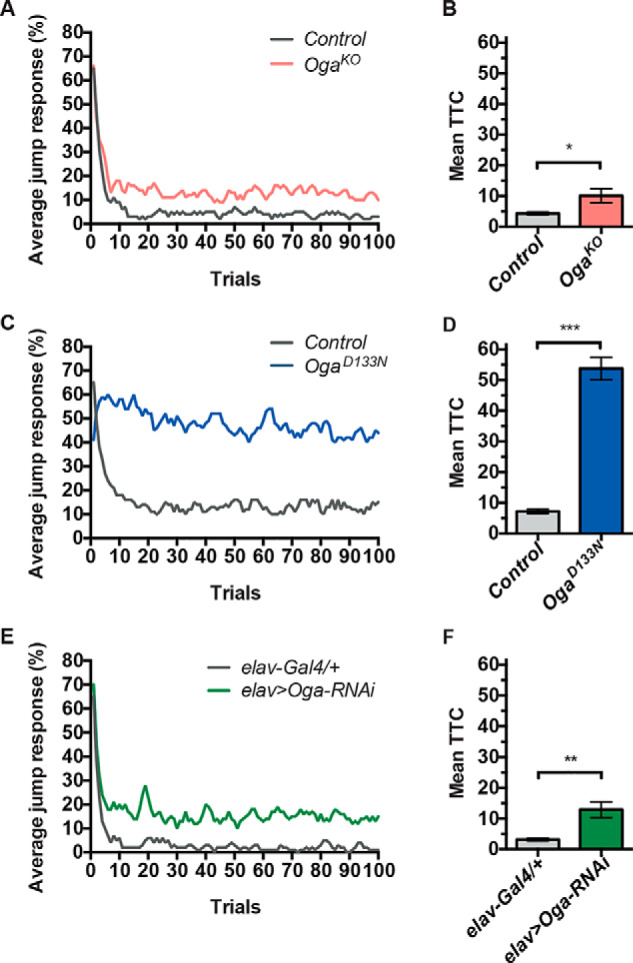
**Loss of Oga activity in *Drosophila* affects nonassociative learning in the light-off jump reflex habituation paradigm.** Jump responses of 3–7-day-old individual male flies were induced by repeated light-off pulses (100 trials) with a 1-s intertrial interval. Habituation was scored as the mean number of trials required to reach the no-jump criterion (TTC). Jump response curves show the average jump response (% of jumping flies) over 100 light-off trials at 1-s intertrial intervals. The number of trials needed to reach the no-jump criterion is presented as mean TTC ± S.E. *A* and *B*, habituation of homozygous *Oga^KO^* male flies (*n* = 63) was significantly slower compared with control flies (*n* = 65) *p* = 0.030. *C* and *D*, habituation of homozygous *Oga^D133N^* male flies (*n* = 86) was significantly slower compared with control flies (*n* = 74) *p* < 0.001. *E* and *F*, habituation of adult flies with neuronal knockdown of Oga (elav::GAL4/+; UAS-Oga^RNAi41822^/+, *n* = 68) was significantly impaired compared with control flies (elav::GAL4/+, *n* = 45; *p* = 0.002).

To dissect a possible role of Oga in cognition, we investigated the effect of *Oga* mutations on habituation. Habituation is a fundamental, evolutionarily conserved form of learning characterized by a temporal attenuation of an initial strong response to a repeated, irrelevant stimulus. It is an important prerequisite for higher cognitive functioning and has been found to be defective in a number of neurodevelopmental disorders in humans (45, 46) and animal models (47–49). To assess the role of Oga and its *O*-GlcNAcase catalytic activity in this type of learning, we subjected *Oga^D133N^* and *Oga^KO^* flies and isogenic genetic background control flies to 100 short (15 ms) light-off stimuli with 1-s intervals in the light-off jump habituation assay. Locomotor deficits observed in the negative geotaxis and island assay did not preclude assessment of habituation, as assessed by a fatigue assay (Fig. S4). All fly lines were able to exhibit good initial jump responses to the first five light-off stimuli (>50% initial jumpers; Tables S1 and S2). Control flies habituated quickly to the repeated light-off stimulus (mean ± S.E. trials to criterion (TTC) = 4.3 ± 0.5, *n* = 65, Fig. 3*A*; and 7.2 ± 0.8, *n* = 74, Fig. 3*B*). Both homozygous *Oga* mutant lines showed slow habituation and failed to adapt their jump response to the repeated stimulus (*Oga^D133N^*: 53.8 ± 3.7, mean TTC fold change = 7.5, *n* = 86; *Oga^KO^*: mean ± S.E. TTC = 10.1 ± 2.3, mean TTC fold change compared with control flies = 2.3, *n* = 63; Fig. 3, *A–D*, and Table S2). We generated and tested also pan-neuronal *Oga* knockdown flies (*elav*>*Oga^RNAi^*), using an inducible *Oga* UAS-RNAi allele (Vienna Drosophila Resource Center (VDRC) catalog no. 41822) and the pan-neuronal elav-Gal4 driver to address the neuronal/glial-specificity of these defects. Progeny from crosses between the *elav-Gal4* driver line and the genetic background of the RNAi line (VDRC catalog no. 60000) were used as controls. The knockdown flies showed good initial jump response (70.8% initial jumpers) but failed to habituate compared with controls (mean ± S.E. TTC = 12.9 ± 2.5, mean TTC fold change = 4.2, *n* = 68; Fig. 3, *E* and *F*, and Table S2). Taken together, these data show that loss of *Drosophila* Oga and its *O*-GlcNAcase activity leads to a deficit in habituation learning and that Oga activity in neurons is required for habituation.

### O-GlcNAcase modulates the number of synaptic boutons at the larval neuromuscular junction

**Figure 4.**
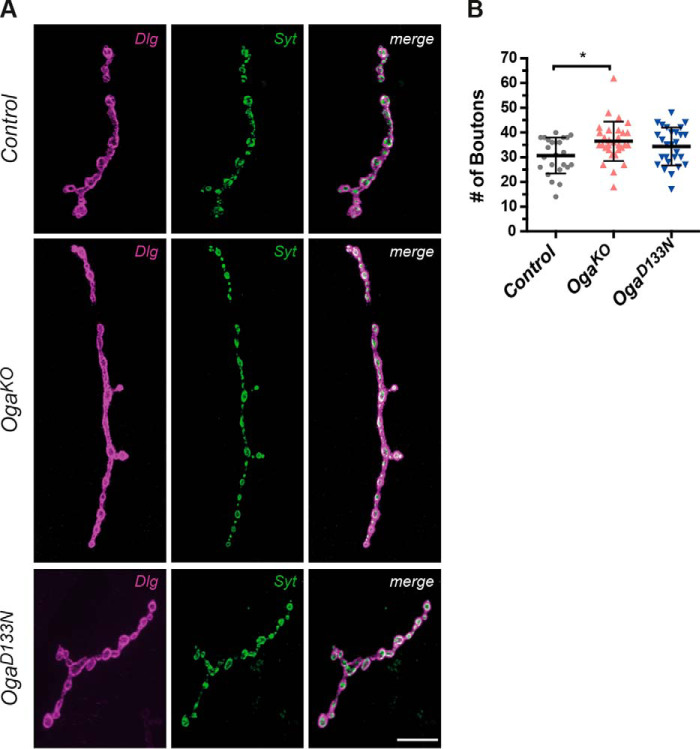
***Drosophila* Oga regulates bouton number in larval NMJ.** Type 1b muscle 4 synapses from wandering third instar larvae were double-stained with anti-discs large 1 (*Dlg*, *magenta*) and anti-synaptotagmin (*Syt*, *green*). *A*, representative NMJs are shown for genetic background control, *Oga^KO^*, and *Oga^D133N^. Scale bar*, 20 μm. *B*, quantification of the total number of boutons based on *Syt* staining indicates an increased number of synaptic boutons present in *Oga^KO^* (*p* = 0.019, *n* = 30). A similar trend was apparent in *Oga^D133N^* (*p* = 0.209, *n* = 27) compared with control (*n* = 24) larvae (one-way ANOVA with Tukey post hoc test). The data are presented as means ± S.D.

Normal synaptic development and morphology are crucial for motor behavior, learning, and cognitive functioning. Importantly, a significant number of proteins that orchestrate synapse structure and synaptic transmission are modified with *O*-GlcNAc (50, 51), including in *Drosophila* (38). We next investigated synaptic morphology in *Oga^D133N^* and *Oga^KO^* flies. The larval neuromuscular junction (NMJ) is a well-established system to study synaptic development and morphology in *Drosophila*. Type 1b NMJs consist of branched chains of synaptic boutons containing glutamatergic transmission sites, which share fundamental mechanistic features with the excitatory system in the mammalian brain (52). We visualized the larval NMJ architecture by immunolabeling the presynaptic marker Syt (synaptotagmin) and Dlg1 (postsynaptic discs large 1) proteins (Fig. 4*A*). Morphometric features of individual muscle four NMJs were quantified semiautomatically (53, 54). We detected mean values of area, perimeter, and length in *Oga^D133N^* and *Oga^KO^* NMJs similar to those in controls (Fig. S5). The numbers of synaptic islands, branches, and branching points were also unaffected in the *Oga* mutants (Fig. S5). However, we observed a modest increase in the number of boutons in *Oga^D133N^* NMJs, which did not reach statistical significance (mean ± S.D., 34.4 ± 7.6), and a significant increase in *Oga^KO^* (36.5 ± 7.9) compared with background control (30.7 ± 7.3) larval NMJs (Fig. 4*B*). These data indicate that Oga potentially influences the number of synaptic boutons at the axon terminals at the larval neuromuscular junction.

## Discussion

Earlier studies have uncovered a link between *O*-GlcNAcase and learning in mouse and rat models (30, 31). Heterozygous *Oga*^+/−^ mice with increased *O*-GlcNAc levels exhibited hippocampal-dependent spatial learning and memory defects (31), whereas rats treated with an OGA inhibitor, thiamet-G, showed reduced performance in novel object and placement tests (30). These learning phenotypes were associated with dysregulation of synaptic plasticity, long-term synaptic potentiation, and α-amino-3-hydroxy-5-methyl-4-isoxazolepropionic acid receptor (AMPAR)–dependent long-term synaptic depression (30). Several *O*-GlcNAc-modified proteins were found that operate at the mammalian synapse, such as Bassoon, Piccolo, and Synapsin (51); regulate transcriptional programs relevant to synaptic plasticity in neurons, such as the cAMP-response element binding protein (CREB) (55); control neuronal microtubule dynamics, such as Tau (56) and CRMP2 (57); or mediate synaptic transmission, such as the AMPAR Glu2 subunit (30). *O*-GlcNAcylation on these and other proteins together potentially modulates neuronal functions. Although the molecular mechanism behind these learning and synaptic phenotypes are not fully understood, our current knowledge indicates that synergic response of multiple voltage-gated ion channels (58) and dysregulation of AMPAR are involved (31, 58).

In contrast to mammalian organisms where MGEA5/OGA is crucial for embryonic development (13, 14), *Oga* is not essential in *Drosophila melanogaster* (15, 16) and *Caenorhabditis elegans* (59). However, the fact that the *Oga* gene is conserved across invertebrates suggests that maintenance of homeostatic *O*-GlcNAc levels by OGA may provide a considerable advantage to Metazoa. Previous work has shown that knockdown of *Oga* in the fly leads to altered metabolism through effects on insulin-producing cells (60, 61). Some of the phenotypes, for example the life-span effects that we observe here (Fig. 2*A*), could be a manifestation of this. However, we also demonstrated that *Oga^KO^* and *Oga^D133N^* mutations led to deficits in habituation, highlighting that the role of OGA in learning is evolutionarily conserved. Our data also provide evidence that the *Drosophila* nervous system is sensitive to an increase of the level of *O*-GlcNAcylation and absence of OGA, establishing it as a suitable genetic model system to study underlying mechanisms and substrates involved.

It has been reported previously that increased protein *O*-GlcNAcylation caused impaired synaptic plasticity in *Oga*^+/−^ mice without affecting dendritic spine density in CA1 pyramidal neurons (31). Here, we report that bouton number of the larval NMJ is affected in *Oga^KO^* null mutants, showing that synaptic morphology is altered in *Oga^KO^* animals.

Although *Oga^D133N^* and *Oga^KO^* caused changes in protein *O*-GlcNAcylation to the same extent, we described behavioral and neuronal phenotypes that manifested to a different degree in the two *Oga* lines. Reduction of total daily activity and an increase in NMJ bouton number was only apparent in *Oga^KO^*, whereas this genotype appeared to exhibit less severe habituation deficits. A possible explanation of this lies in the choice of inactivating mutation. Previous work in mammalian and bacterial *O*-GlcNAcases has shown that the equivalent of the D133N mutation inactivates the enzyme. However, recent work has uncovered that this mutation does not lose the ability to bind *O*-GlcNAc proteins; indeed this inactive mutant can be used to enrich the *O*-GlcNAc proteome (38, 62). Therefore, it is possible that the D133N mutation contributes to the stronger habituation phenotype by binding to (parts of) the *O*-GlcNAc proteome in the fly, interfering with *O*-GlcNAc signaling/sites. Thus the *Oga^D133N^* potentially behaves as a neomorphic allele. It is possible that the absence or presence of the Oga protein affects the phenotypes. For example, the reduction in daily activity and an increase in NMJ bouton number specific for the *Oga^KO^* allele might emerge as a combined effect of lack of Oga activity and absence of the Oga protein. Our results thus suggest that such additional functions could modulate the phenotypes arising from a complete loss of *O*-GlcNAcase activity.

In summary, we have shown that *Oga* regulates *O*-GlcNAc homeostasis, thus influencing life span, locomotor, and neuronal performance in *D. melanogaster*. Further studies are required to define the mechanisms downstream of *Oga* that affect neuronal development or function, resulting in synaptic morphology and habituation learning defects.

## Experimental procedures

### Cloning of the guide RNA and repair template DNA vectors for Drosophila CRISPR/Cas9 editing

*D. melanogaster* lines lacking Oga activity, *Oga^KO^* and *Oga^D133N^*, were generated using the CRISPR/Cas9 gene-editing technique following a workflow as previously described (74). A guide RNA site was selected with the help of the CRISPR online tool search, and the annealing primer pair (gRNA_oga_fwd and gRNA_oga_rev) with appropriate overhangs for BpiI restriction digestion was cloned into pCFD3-dU63gRNA plasmid (63). A vector coding for repair template DNA of 2044 base pairs was generated from *Drosophila* Schneider 2 cell genomic DNA by PCR using GoTaq G2 polymerase (Promega), A1fix_BAM_fwd, and A1fix_NOT_rev primers. The PCR product was inserted into pGEX6P1 plasmid. The desired mutation, in addition to five silent mutations (Fig. S2), was introduced by site-directed mutagenesis using the paired primers D133N wobble F and R. The silent mutations removed a neighboring *Taq*^α^*I* restriction site, thus enabling genotyping based on restriction digestion. DNA products of cloning and mutagenesis were confirmed by sequencing. All primer sequences are listed in Table S2.

### Generation of Oga^D133N^ and Oga^KO^ Drosophila lines

*Drosophila* embryos expressing Cas9 in the germ-line cells (vasa::Cas9, Bloomington stock no. 51323) were injected with a mixture of CRISPR/Cas9 reagents, 100 ng/μl guide RNA plasmid, and 300 ng/μl repair template DNA vector. Microinjections were performed at the University of Cambridge fly facility. Founder male flies were crossed with *Dr/Tm6* balancer stock in-house. Subsequently, single potential *Oga^D133N^/Tm6* germ-line mutant male flies and *Dr/Tm6* virgins were crossed that allowed for elimination of the *vasa::Cas9* carrying X chromosome. Candidate F1 males were genotyped exploiting restriction fragment length polymorphism arising from the loss of *Taq*^α^*I* restriction digestion site introduced in parallel with the D133N mutation (Fig. S2*A*). We recovered a knockin line carrying the precise mutation, *Oga^D133N^*, and a knockout line where a frameshift resulting from nonhomologous end joining repair of a CRISPR event introduced a premature STOP codon into the *Oga* sequence, *Oga^KO^* (Fig. S2*B*). Lines were validated first by sequencing the diagnostic digest PCR product confirming the region ∼250 base pairs upstream and downstream of the mutation. This was followed by production of larger PCR products encompassing an area outside the repair template. Sequencing of these products confirmed that only the intended changes were introduced to *Oga* and excluded the possibility of ectopic integration of the repair template somewhere else in the genome. To eliminate any potential off-target mutations introduced during CRISPR, our *Oga* lines were backcrossed into the *w*^1118^ control genetic background for six generations prior to experimentation.

### Restriction fragment length polymorphism assay for genotyping Oga^D133N^ and Oga^KO^ lines

To assess and confirm presence of the D133N and KO mutation in the *Oga* gene, candidate individual adult flies were frozen and homogenized in 10–50 μl of DNA extraction buffer containing 10 mm Tris-HCl, pH 8, 1 mm EDTA, 25 mm NaCl, and 200 μg/ml freshly added proteinase K (Roche) and subsequently incubated at 37 °C for 30 min, followed by inactivation of proteinase K at 95 °C for 3 min, and centrifuged briefly. 1 μl of the crude DNA extract was used per 25-μl PCR with A1_DIG F and R primers and KOD Hot start polymerase (Novagen). 5 μl of the 589-bp PCR product was used for restriction fragment length polymorphism assay with Taq^α^I followed by agarose gel electrophoresis of the digested products. Full-length fragments resistant to Taq^α^I cleavage indicated a CRISPR/Cas9 gene editing event and were sequenced using A1_DIG primers. Precise incorporation of the repair template into the right position of the genome was confirmed by sequencing a second round of PCR products obtained from potential homozygous CRISPR mutants with mixed A1_DIG and A1_OOB primer pairs. The primer sequences are listed in Table S2.

### Fly stocks and maintenance

All *Drosophila* strains and crosses were reared on a standard *Drosophila* diet (sugar/cornmeal/yeast). Unless stated otherwise, all crosses were raised at 25 °C, 70% humidity, and 12:12-h light–dark cycle. RNAi strain against *Drosophila Oga* (no. 41822, zero predicted off-targets, >60% reported knockdown efficiency (60), and a control strain (no. 60000) were obtained from the VDRC.

*Oga^D133N^* and *Oga^KO^* strains were crossed into the VDRC *w*^1118^ control genetic background for six generations. This isogenic background strain was used as control for experiments on *Oga^D133N^* and *Oga^KO^* strains. The *w*^1118^; *2xGMR-wIR*; *elav-Gal4*, *UAS-Dicer-2* driver strain was used to induce neuronal knockdown. This strain contains a double insertion of an RNAi construct targeting the gene *white* specifically in the *Drosophila* eye (*2xGMR-wIR*) to suppress pigmentation, as required for an efficient jump response in light-off jump habituation (49, 64, 65). Progeny of the cross between the RNAi strain and the respective genetic background were tested in habituation experiments.

### Western blotting from Drosophila samples

To prepare protein lysates for Western blotting, homozygous parental flies were set up in egg-laying cups with apple juice plates at 25 °C. 4–8-h embryos were collected, dechorionated with bleach, and snap-frozen in liquid nitrogen. Also, 1–4-day-old adult male flies were collected and snap-frozen in liquid nitrogen. Heterozygous flies were obtained from crossing *Oga^KO^* and *Oga^D133N^* with *w*^1118^. Heads were separated by vigorous vortexing for 2 × 15 s. The frozen samples were homogenized in 2 μl/1 head lysis buffer containing 2× NuPAGE LDS sample buffer, 50 mm Tris-HCl (pH 8.0), 150 mm NaCl, 4 mm sodium pyrophosphate, 1 mm EDTA, 1 mm benzamidine, 0.2 mm phenylmethylsulfonyl fluoride, 5 μm leupeptin, and 1% 2-mercaptoethanol. The lysates were then heated for 5 min at 95 °C and centrifuged at 13,000 rpm for 10 min, and the supernatants were collected. Protein concentrations were estimated using the Pierce 660-nm protein assay supplemented with ionic detergent compatibility reagent (Thermo Scientific). Protein concentrations were adjusted across samples. 20–30 μg of protein lysate was loaded on NuPAGE 4–12% Bis-Tris protein gels (Invitrogen) and transferred onto nitrocellulose membrane. The membranes were developed with mouse anti-*O*-GlcNAc antibody, RL2 (1:1000, Thermo), rabbit anti-OGT (1:1000, Abcam, ab-96718), rabbit anti-OGA (1:1000, Sigma, SAB4200267), and rabbit anti-actin (1:5000, Sigma, A2066) primary antibodies and donkey anti-mouse IgG 800 and goat anti-rabbit IgG 680 IR dye–conjugated secondary antibodies (Li-Cor, 1: 10000). Western blots were recorded on a LiCOR system, and signal was measured using Lite software. Significance was calculated using one-way ANOVA with Dunnett's multiple comparisons test.

### Measurement of life span

20 age-matched male flies were placed in a vial within 24 h of eclosion for recording their life span. The flies were flipped in every 2–3 days, and the number of flies alive were recorded. At least 9 vials (control, 9 vials; *Oga^KO^*, 14 vials; and *Oga^D133N^*, 14 vials) for each genotype were tracked. The vials were kept in the same tray and incubator at 25 °C and 12:12-h light-dark cycle. A log-rank test was used to compare life spans across genotypes.

### Drosophila activity monitoring

*Drosophila* locomotor activity was monitored using the DAM2 *Drosophila* activity monitoring system (TriKinetics) at 25 °C and 12:12-h light-dark cycle, using 2–3-day-old male flies. The method was described in detail previously (66). Food mixture containing 2% agar, 5% sucrose was loaded to one end of 65-mm tubes. Activity was recorded for 5 days, and the data obtained on the 2nd to 5th days were used and merged for this study. The data sets were analyzed using the Sleep and Circadian Analysis MATLAB Program (S.C.A.M.P.) (67). Combined data from four independent measurements with 20–30 flies per genotype was analyzed; statistical significance was calculated using one-way ANOVA with Bonferroni's multiple comparisons test.

### Island assay

The island assay was used to evaluate the flight locomotor behavior of the 2–3-day-old male flies as described previously (68, 69). 15 flies from each genotype were subjected to the assay per run. 3–4 repeats were carried out on each day, and the data were collected on 3 consecutive days; in total, we collected data from 9–12 runs per genotype. AUC was determined for each run; groups were compared using ANOVA with Holm–Sidak's multiple comparisons of means for AUC.

### Negative geotaxis test

Climbing assay was performed as described previously (43). Briefly, 0–2-day-old male flies were collected and divided into groups of 10 animals at least 48 h before the measurement. Climbing ability of 3–6-day-old flies was evaluated. On the day of the measurement, the flies were transferred into 150 × 16-mm transparent plastic test tubes without anesthesia. A maximum of 10 test tubes were placed into a frame that allowed for monitoring of climbing behavior of up to 100 animals at once. The frame was secured onto an apparatus that releases the frame from a fixed height upon pushing a button. The frame falls down onto a mouse pad, thereby tapping the flies to the bottom of the tubes. The climbing assay was repeated four times for each loaded frame, providing data from four runs. The whole procedure was recorded with a Nikon D3100 DSLR camera. The resulting movies were then analyzed with ImageJ/Fiji software. First, the images were converted to 8-bit gray-scale TIFF image sequence (10 frames per second) file format. Following background subtraction and filtering, the image sequences were binarized to allow for tracking of flies using the MTrack3 plug-in. Mean climbing speed (mm/s) was quantified for each genotype in each run; between 19 and 89 data points were collected per run. The groups were compared using Mann–Whitney test on mean climbing speed values calculated for each run.

### Light-off jump habituation

The light-off jump reflex habituation assay was performed as previously described (49, 70). Briefly, 32 individual 3–7-day-old male flies were transferred in the habituation chambers of two independent 16-unit light-off jump habituation systems. Male progeny of the control genetic background were tested simultaneously and served as controls in all experiments. The flies were tested at 25 °C and 70% humidity. They were exposed to a series of 100 light-off stimuli (stimulus duration was 15 ms) with 1-s interstimulus intervals. The noise amplitude of wing vibration accompanying the jump response was recorded after the start of each stimulus. An automatic threshold was applied to distinguish the jump responses from background noise. The data were collected by custom-made Labview software (National Instruments). High initial jump responses to light-off stimulus decreased with the repetition of the stimuli, and the flies were deemed habituated when they failed to jump in five consecutive trials (no-jump criterion). Habituation was quantified as the number of trials required to reach the no-jump criterion (TTC). All experiments were done in triplicate (*n* = 96 flies). Despite reduced locomotion abilities of *Oga^KO^* and *Oga^D133N^* observed in the island and climbing assays, these mutant genotypes exhibited sufficient initial jump responses to the light-off stimuli to allow habituation to be assessed (>50% initial jumpers; Table S2). The main effects of genotype on log-transformed TTC values were tested using a linear model regression analysis (lm) in the R statistical software (R version 3.0.0, April 3, 2013) (71) and corrected for the effects of testing day and habituation system.

### Fatigue assay

The assay measures the ability of flies to perform the jumping task repeatedly for prolonged time. This test was carried out subsequently after the habitation assay on the same flies with each genotype as described previously (49). Jump response is induced with a light-off pulse; however, the interval time between light-off pulses was adjusted to 5 s, a long enough period to prevent the formation of habitation response. The light-off stimuli were repeated 50 times. The jump responses of mutant groups were compared with the appropriate control genotype. The flies that maintained similar jump response to control groups over the 50 trails were interpreted as fit for the habituation assay.

### Analysis of synaptic morphology

Crosses for all mutants and controls were set up with five 1–3-day-old female virgin and male flies (5 and 10 flies, respectively). The adults were flipped out after 36 h from the vials. This step prevented larval crowding and ensured proper staging and equal size of the larvae. The size of the larvae was also examined by the experimenter prior to dissection. Wandering male L3 larvae were dissected with an open book preparation (72) and fixed in 3.7% paraformaldehyde for 30 min. Larvae were stained overnight at 4 °C with the primary antibodies against the following synaptic markers: Discs large (anti-dlg1, mouse, 1:25, Developmental Studies Hybridoma Bank) and synaptotagmin (anti-Syt, rabbit, 1:2000, kindly provided by H. Bellen). Then the samples were stained with secondary antibodies (1:500) anti-mouse Alexa 488 and anti-rabbit Alexa 568 (Invitrogen) for 2 h at room temperature. Projections of type 1b NMJs at muscle 4 from abdominal segments A2–A4 were visualized with Zeiss Axio Imager Z2 microscope with Apotome. Individual synapses were imaged and quantified using an in-house developed macro (53, 54) in Fiji (version 1.49) (73). NMJ area, length, number of branches, and number of branching points were analyzed based on discs large labeling, and the number of synaptic boutons was analyzed based on synaptotagmin labeling. Parameters with normal distribution (area, length, and number of boutons) were compared between the mutants and control with one-way ANOVA with Tukey's multiple comparison test. Parameters without normal distribution (number of branches and branching points) were compared with nonparametric Wilcoxon test. Statistical analysis was carried out in SPSS.

## Author contributions

V. M., M. F., and D. M. F. v. A. conceptualization; V. M. and M. F. data curation; V. M., M. F., M. C., and I. E. formal analysis; V. M., M. F., and M. C. investigation; V. M. visualization; V. M., M. F., A. S., and D. M. F. v. A. writing-original draft; V. M., M. F., I. E., E. S., A. S., and D. M. F. v. A. writing-review and editing; M. F., A. T. F., and I. E. methodology; E. S., A. S., and D. M. F. v. A. funding acquisition; A. S. and D. M. F. v. A. resources; A. S. and D. M. F. v. A. supervision; A. S. and D. M. F. v. A. project administration.

## Supplementary Material

Supporting Information

## Footnotes

3 The abbreviations used are:

OGT: *O*-GlcNAc transferase
OGA: *O*-GlcNAcase
AUC: area under the curve
TTC: trials to criterion
VDRC: Vienna Drosophila Resource Center
NMJ: neuromuscular junction
AMPAR: AMPA receptor
ANOVA: analysis of variance.
