# Supplementary material for: O-GlcNAcase contributes to cognitive function in Drosophila
Source: J Biol Chem. 2020 Feb 24;295(26):8636–46. doi: 10.1074/jbc.RA119.010312 (PMC7324509; doi:10.1074/jbc.RA119.010312)
Supplement: Supporting Information [file supp_295_26_8636__index.html]

O-GlcNAcase contributes to cognitive function in Drosophila — Drosophila Oga is required for habituation learning — O-GlcNAcase contributes to cognitive function in Drosophila — Drosophila Oga is required for habituation learning — Supporting Information 

# *O*-GlcNAcase contributes to cognitive function in *Drosophila*

## Supporting Information

- Supplementary Figures and Tables - Figure 1 Sequence alignment of CpOGA, hOGA and DmOga. Figure 2 CRISPR strategy and genotyping of OgaD133N and OgaKO Drosophila lines. Figure 3 Daily activity behaviour of OgaD133N and OgaKO Drosophila. Figure 4 Fatigue assay tests motor function required for the habituation task. Figure 5 Quantification of NMJ parameters in OgaKO and OgaD133N Drosophila larvae, based on Dlg staining. Supplementary Table S1. Primers used for cloning CRISPR/Cas9 reagents and genotyping OgaKO and OgaD133N Drosophila lines. Supplementary Table S2. Habituation parameters are shown for combined datasets obtained over three independent measurements.
