# Supplementary material for: O-GlcNAcase contributes to cognitive function in Drosophila
Source: J Biol Chem. 2020 Feb 24;295(26):8636–46. doi: 10.1074/jbc.RA119.010312 (PMC7324509; doi:10.1074/jbc.RA119.010312)
Supplement: Supporting Information [file supp_RA119.010312_154664_2_supp_479908_q67h74.pdf]

Supporting Information

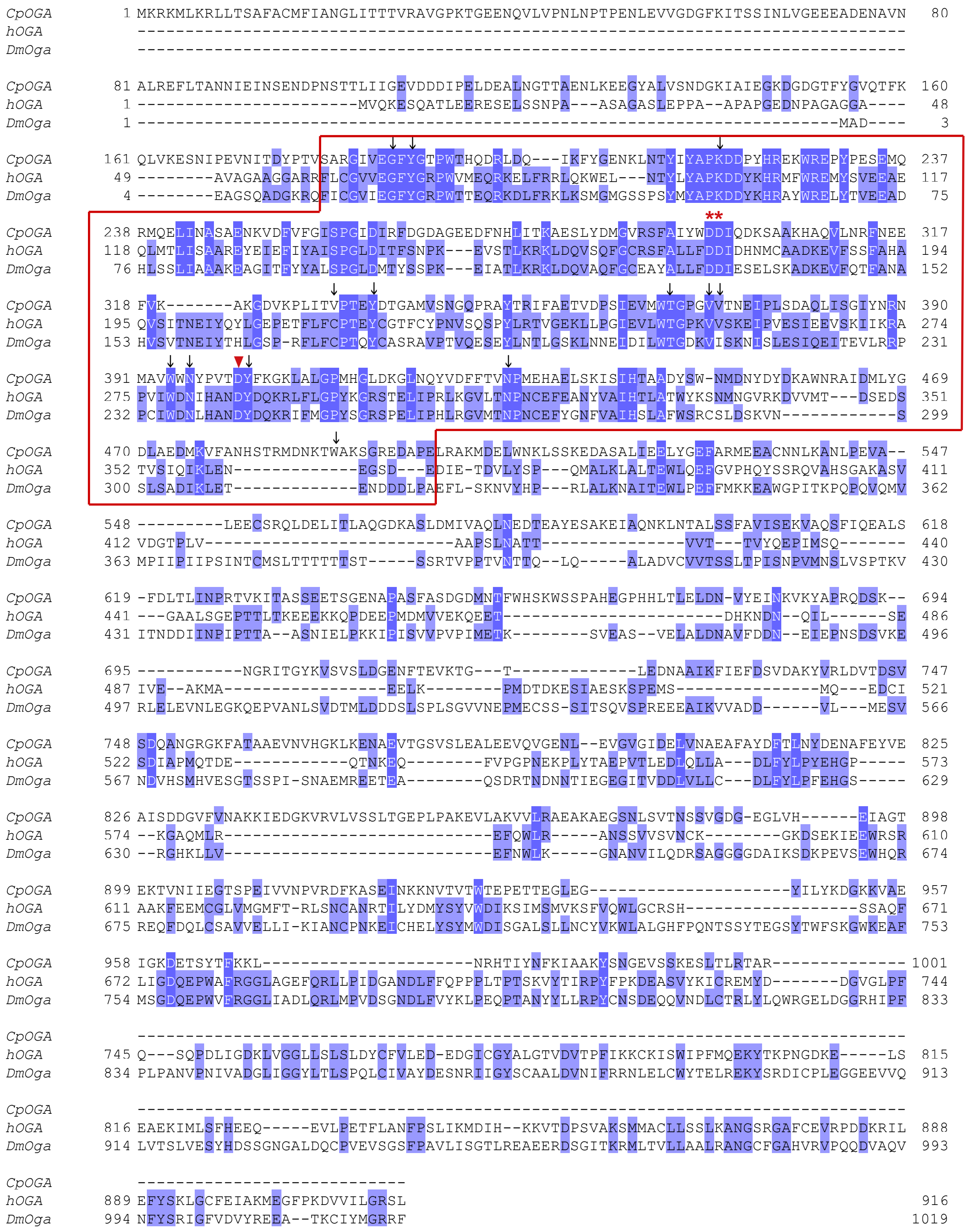

Supplementary Figure S1. Sequence alignment of *Cp*OGA, *h*OGA and *Dm*Oga. O-GlcNAcase catalytic domain is shown in a red box. Residues of the active site are marked with an arrow. Amino acids of the catalytic pocket that are essential for catalytic activity are marked with red *, and residues required for O-GlcNAc binding are indicated with red ▽ (35). Sequeces were obtained from Uniprot, Q0TR53, O60502 and Q9VDC9, respectively. Sequences were aligned with Jalview 2.11.0 and identical amino acids are highlighted in blue.

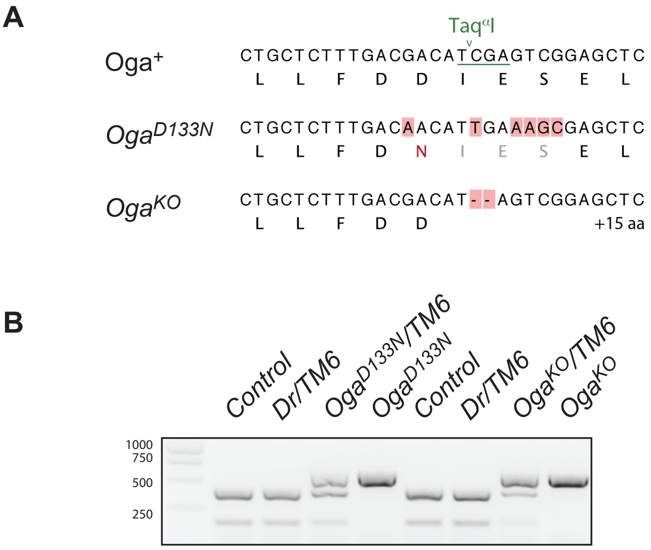

Supplementary Figure S2. CRISPR strategy and genotyping of *Oga^{D133N}* and *Oga^{KO}* *Drosophila* lines. (**A**) Sequences of genomic DNA of wild type, *Oga^{D133N}* and *Oga^{KO}* *Drosophila* alleles. For screening purposes, the endogenous TaqαI restriction site was also abolished by targeted mutagenesis in parallel with introducing the D133N mutation. Furthermore, four silent mutations were incorporated to create wobble sites adjacent to the D133N mutation. *Oga^{KO}* was generated through imperfect CRISPR/Cas9 editing introducing two nucleotides frameshift that resulted in early termination codon. Red highlight indicates changes in the genomic DNA and protein sequence.

(**B**) Representative agarose gel demonstrates genotyping based on restriction fragment length polymorphism after TaqαI restriction of PCR fragment, that allowed for distinguishing between wild type, heterozygous and homozygous *Oga^{D133N}* and *Oga^{KO}* flies.

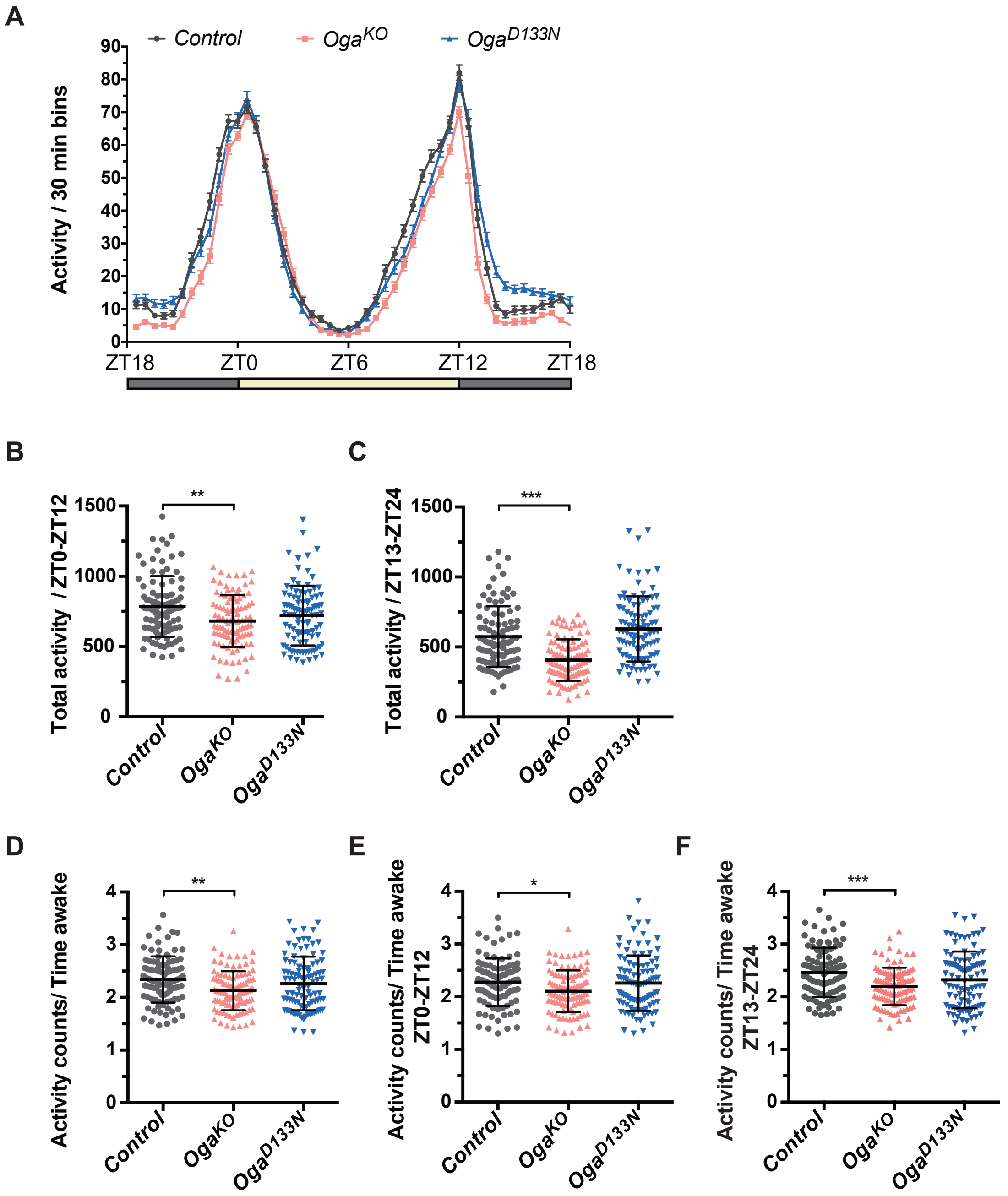

Supplementary Figure S3. Daily activity behaviour of *Oga^{D133N}* and *Oga^{KO} Drosophila.* (**A**) Histogram showing daily locomotor activity profiles of Control (n = 97), *Oga^{KO}* (n = 101) and *Oga^{D133N}* (n = 98) male *Drosophila* in 12:12 h light:dark cycle. X axis - Zeitgeber Time (ZT). Data shown here resulted from averaging the second to the fifth days of the recordings, generating the 24 h profiles shown in the panel, mean ± SEM. (**B**) Total activity count in light phase, mean ± SD, *Oga^{KO}* exhibited a decrease in activity compared to Control flies (***p* = 0.0012, one-way ANOVA with Bonferroni's multiple comparisons test) (**C**) Total activity count in the dark phase, mean ± SD. *Oga^{KO}* exhibited a decrease in activity compared to Control flies (****p* < 0.0001, one-way ANOVA with Bonferroni's multiple comparisons test) (**D**)-(**F**) Activity counts/ time awake parameter is decreased in *Oga^{KO}* compared to Control flies, mean ± SD (**p* = 0.0264, ***p* = 0.0026, ****p* = 0.0002, one-way ANOVA with Bonferroni's multiple comparisons test).

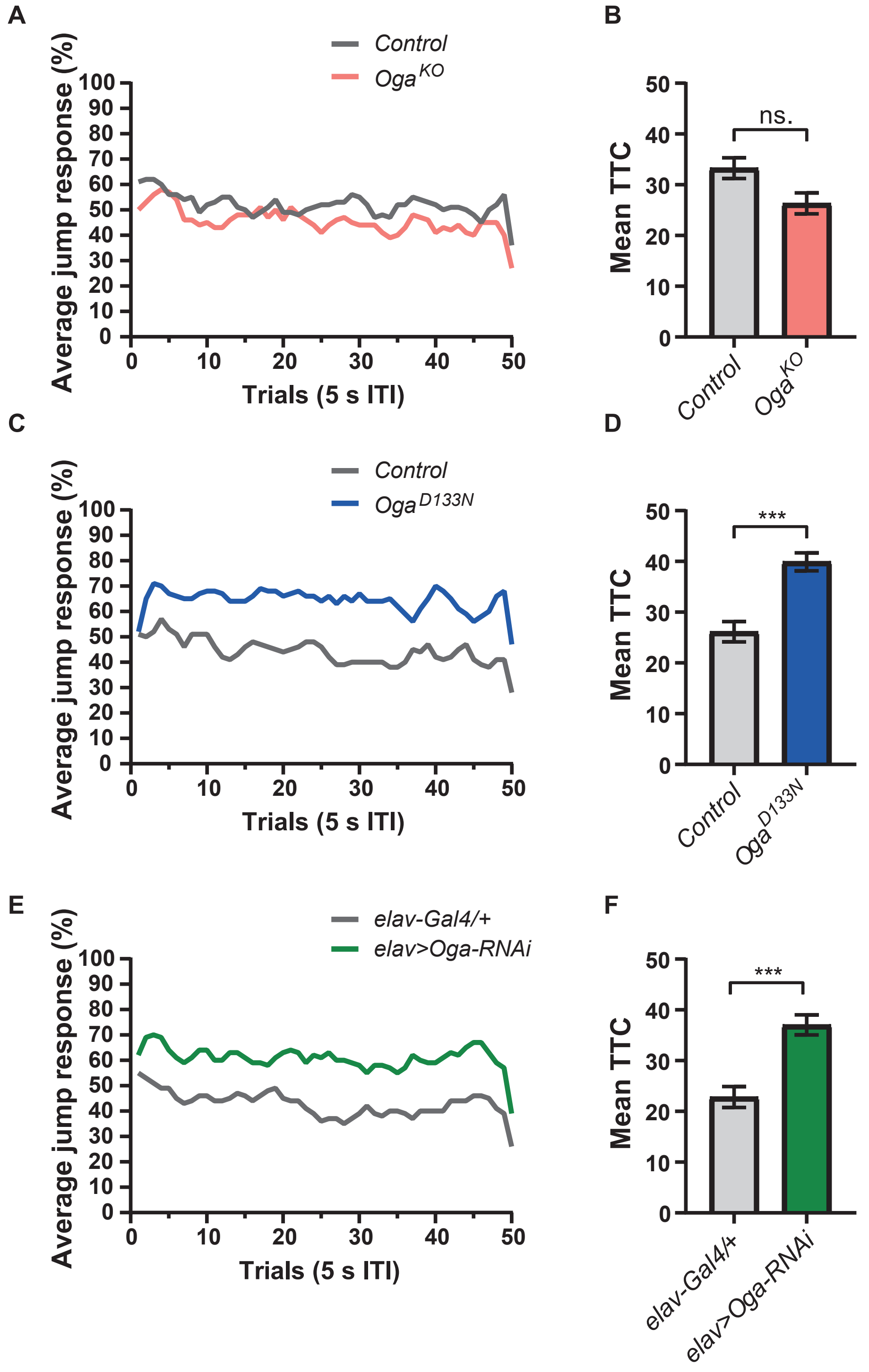

Supplementary Figure S4. Fatigue assay tests motor function required for the habituation task. Jump responses of 3–7-day-old individual male flies were induced by repeated light-off pulses (50 trials) with a 5 s inter-trial interval (ITI), a period sufficiently long to prevent the formation of habituation response, so healthy flies keep jumping. Jump response curves show the average jump response (% of jumping flies) for each trial. The number of trials needed to reach the no-jump criterion is presented as Mean TTC ± SEM. (**A**) and (**B**) Fatigue assay indicated similar jump response of homozygous Oga^{KO} male flies (n = 76) compared to control flies (n = 86) $p = 0.074$. (**C**) and (**D**) Fatigue assay showed significantly higher jump response in Oga^{D133N} male flies (n = 90) compared to control flies (n = 84) $p < 0.001$. (**E**) and (**F**) Fatigue assay suggested that jump response of adult flies with knockdown of *Oga*, (*elav::-GAL4/+; UAS-OgaRNAi*$^{41822/+}$, n = 85) was significantly higher compared to control flies (*elav::-GAL4/+*, n = 81) $p < 0.001$.

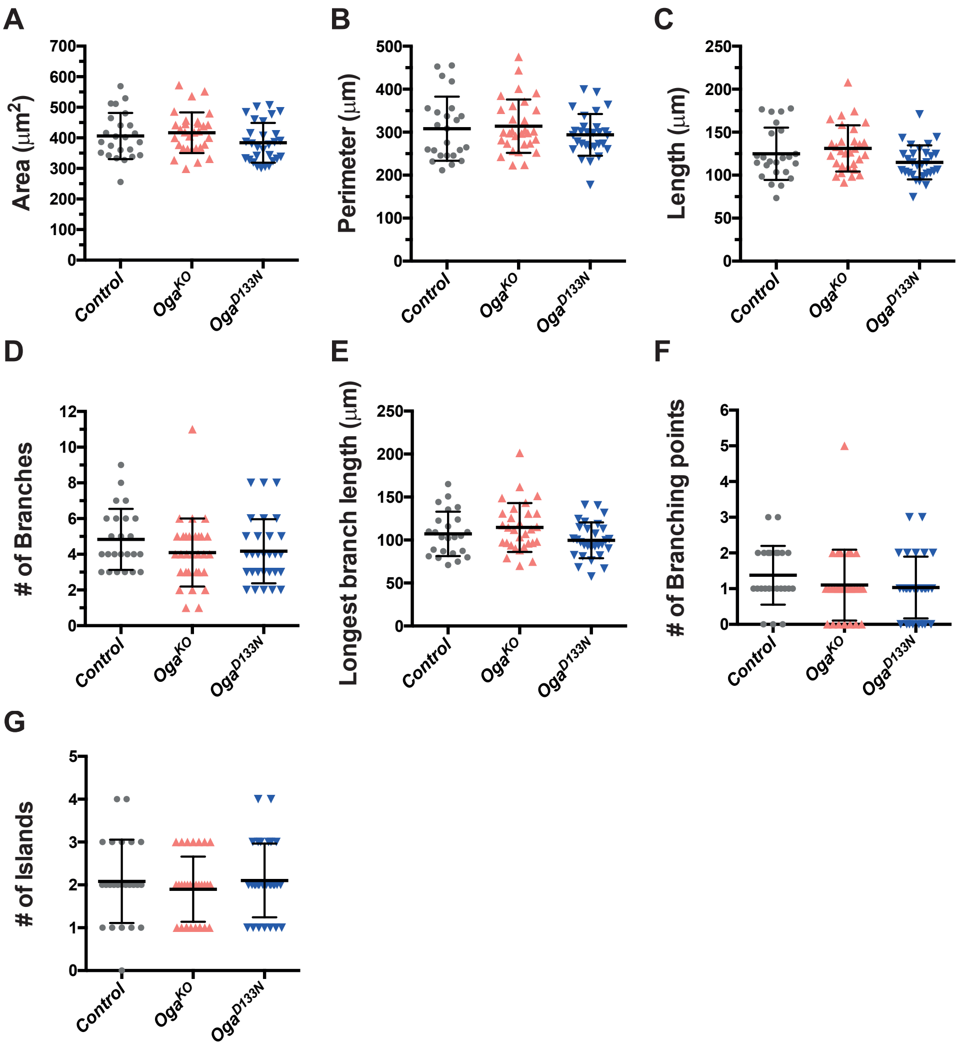

Supplementary Figure S5. Quantification of NMJ parameters in *Oga^KO^* and *Oga^D133N^ Drosophila* larvae, based on Dlg staining. (**A**) Area. (**B**) Perimeter of NMJs. (**C**) Total NMJ length. (**D**) Number of NMJ branches. (**E**) Longest branch length. (**F**) Number of NMJ branching points. (**G**) Number of NMJ islands.

**Guide RNA**	CF_DmA133_fwd	GTCGCTCTTTGACGACATCGAGT
**Guide RNA**	CF_DmA133_rev	AAACACTCGATGTCGTCAAAGAG
**Genomic DNA**	A1fix_BAM_fwd	aaaGGATCCGGATAGTATGGTGACGGGACTTGACC
**Genomic DNA**	A1fix_NOT_rev	tttGCGGCCGCTCACTTTTGTGGGTGAGACCAGGGAG
**Mutagenesis**	D133N_wobbleF	GCCTACGCCCTGCTCTTTGACaACATtGAaagcGAGCTCTCAAAGGCGGACAAG
**Mutagenesis**	D133N_wobbleR	CTTGTCCGCCTTTGAGAGCTCgcttTCaATGTtGTCAAAGAGCAGGGCGTAGGC
**Genotyping**	A1_DIG_F	CCGATGGCAAGCGGCAGTTTATCTG
**Genotyping**	A1_DIG_R	ATTCCTGGACCGTTGGCACCGCTC
**Line check**	A1_OOB_F	CGCGATCATCGCACGTCCCTGATG
**Line check**	A1_OOB_R	ATGGGATTGATGATGTCATCGTTCGTG
**Line check**	A1_seq1	GGCAAGCGGCAGTTTATCTG
**Line check**	A1_seq2	ACCCTGGGCTCCAAGCTG

Supplementary Table S1. Primers used for cloning CRISPR/Cas9 reagents and genotyping *Oga*^{KO} and *Oga*^{D133N} *Drosophila* lines.

Condition	N total	N jumpers	% of Jumpers	Mean TTC	Mean TTC Sd	Mean TTC SEM	Mean TTC fold change	*p* value
Control	96	65	68	4.3	4.0	0.50		
Oga^{KO}	96	63	66	10.1	18.1	2.28	2.3	0.0296
Control	96	74	77	7.2	6.8	0.79		
Oga^{D133N}	96	86	90	53.8	34.7	3.74	7.5	<0.0001
elav>>Gal4	96	45	47	3.1	2.9	0.43		
elav::OgaRNAi^{41822}	96	68	71	12.9	21.0	2.54	4.2	0.0017

Supplementary Table S2. Habituation parameters are shown for combined datasets obtained over three independent measurements.
